# Rachel Challice's Vow

**Published:** 1888-03-03

**Authors:** Lina Nevill

**Affiliations:** Author of "A Future on Trust," "Juno," &c.


### Rachel Challice's Vow.

By Lina Nevill, Author of "A Future on Trust," "Juno," &c.

### Chapter XI.—Doubt.

"I— the same sad prospect find,
And wake to all the griefs I left behind."—*Pope.*

Now that Nell was so far advanced towards complete recovery, the next question was getting her away for change.

She longed to be out of London. Dr. Graves gave his permission; indeed, a thorough change, he said, would be the best thing now for her; just what she wanted to set her up after the confinement and forced inactivity of the last few months.

With all England, Scotland, and Ireland, to say nothing of the Continent, before them, the difficulty seemed to be to fix upon a place which they all three agreed in liking.

Mrs. Endsleigh desired a little society if possible. Charley hankered after opportunities of sport. Nell wished to please him, and on her own account go to a place that she had never seen before.

One thing was clear—wherever they went, there they must stay. Nell would not be strong enough to bear constant moving from place to place. The first locality decided upon must be the last also. There could be none of that pleasant, desultory rambling from town to town, or village to village. Even should they find their selected resting-place undesirable in many ways, except they were driven away by sheer discomfort, they would have to make the best of a bad job; and, like children, "try to make believe very hard" that they were thoroughly contented and happy.

"I leave it entirely to you two to decide where we shall go," said Mrs. Endsleigh; "but a place must be definitely fixed upon, and proper accommodation secured, before I allow Nell to move from Grosvenor Square. In fact, before finally settling, I must make it a condition that Charley goes down first and sees that everything is fit for our reception."

"I'll do that willingly," replied Sir Charles.

A knock sounded at the door.

"Come in," cried Nell. "Oh, here is my stern keeper come to say that it is high time you two frivolous people went, and that this child was put to bed. Nurse, we cannot decide where to go for change; we all want something different. Cannot you help us with a suggestion? Where did you live before you came to London?"

"In Wales," replied Rachel, in a low voice.

"The very thing!" exclaimed Nell joyfully. "I have never been in Wales at all. Was it in north or south?"

"North, Miss Endsleigh."

"If you decide on Wales, Nell, go to Bedd-gelert," broke in Charley. "I was there once for a fortnight. Lovely scenery, capital hotel, no end of fishing, and a little rough shooting. I know a man down there who will give me any permission I want. I don't think you could do better if you tried all England over."

"Then Bedd-gelert it shall be," announced Nell decisively. "Good night, Charley," she continued. "You must go now. Come in as early as you can to-morrow, then we will talk over plans, and Nurse Challice shall give us her advice. By-the-by, Nurse, do you know this place? Have you ever been there?"

"Yes, Miss Endsleigh," replied Rachel, almost inaudibly. "I have been to Bedd-gelert."

Rachel went to her room that night with a mind distracted with doubt and fear.

With horror-struck dismay she had heard Nell fix upon Bedd-gelert as their autumn destination, and realised on the instant what such a move must mean for her.

She had promised to accompany the Endsleighs. Nell would not hear of parting with her at present; and Mrs. Endsleigh had implored her, as a personal favour, to come with them wherever they went. The matron's leave had been obtained; and now Nurse Challice forming one of the party was considered a settled thing, a regular matter of course. ate seemed dead against Rachel. The fickle goddess, with her capricious likes and dislikes, seemed to have singled out the poor girl as a special object on which to expend her spite.

The more Rachel thought over the matter, the more determined she became that nothing should induce her to go to Bedd-gelert.

"These Endsleighs had no real claim on her," she argued inwardly, trying against her own convictions to satisfy her mind that she was only dealing fairly by herself, and that she was the sole person concerned—a conviction her naturally unselfish nature refused to concede to. "She was only a paid servant of theirs for the time; free, like any other menial, to throw up her place and shift for herself." It was false argument, unsound theory, and Rachel in her heart knew it to be so.

All the next morning she tried to seize an opportunity to tell Mrs. Endsleigh or Nell that she could not accompany them to Bedd-gelert.

As is often the case when on the watch for such occasions, no possible opportunity seemed to present itself.

Mrs. Endsleigh went out shopping directly after breakfast, resolved to lay in stores of thick boots, ulsters, tailor-made costumes of the most enduring fabric, and hats warranted to withstand the roughest wear. She meant to grapple manfully with the discomforts of her future position, and on all points, as far as possible, be prepared for the worst.

A girl friend, who was passing through town, came unexpectedly to see Nell. This Miss Gordon was to be Nell's chief bridesmaid, so that the two girls found more than enough to talk about in choosing and settling what dress and what colour should be worn at the wedding, and discussing the weighty question of hats or bonnets.

Then, when Miss Gordon left, and Rachel thought she saw her opportunity, Nell was so worn out by the morning's talking and excitement as to be only fit to lie down and keep quite quiet until Sir Charles came about tea-time.

Then there was more discussion of plans, Charley lauding Bedd-gelert to the skies, promising to go down directly and get everything straight for them; Nell lying on her couch, listening to and agreeing with all he said, entering into all his plans for fishing, shooting, and showing her the neighbourhood; Mrs. Endsleigh, calm and resigned, busily knitting away at the white fluffy shawl she was making, and smiling indulgently at the mad plans and wild ideas suggested by her two young companions.

"You will wait until you are married to make that excursion, if you please, Nell," she observed once, when Charley proposed taking Nell up Snowdon, if not too late in the season, a proposal eagerly assented to by the girl. "Once you are in Charley's keeping he may do what he likes with you—you will be his lawful property, to be disposed of as he pleases; but so long as you are under my authority I shall utterly forbid any of these mad excursions. I am not going to run the risk of having you laid up again for months in this dreadful Welsh wilderness to which we are going."

Nell burst out laughing at her mother's despondent tone.

"Poor, darling mother! I do believe you think we are going to carry you off to a barbarous country, where people are clothed in skins, and live on roots and water. The very name of Wales seems to suggest to your mind a sort of savage Ultima Thule. Nurse Challice," as Rachel came in with a letter, "do you relieve poor Mrs. Endsleigh's mind. You said you knew Bedd-gelert. Tell her the people are civilised there, and behave as rational beings, and that a shop and a doctor are not unattainable luxuries."

"There is a very good doctor at Portmadoc; that is only eight miles away," replied Rachel, accustomed to counting distances, and thinking little of the one she mentioned.

"Eight miles!" ejaculated Mrs. Endsleigh. "A day's journey! And the railway, how near does that come? how far shall we be from the station?"

"Rhyd-ddu will be the nearest; that is only three or four miles from Bedd-gelert. You will go there coming from London, not to Portmadoc."

"This is all worse than I expected," said Mrs. Endsleigh sorrowfully.

"Oh, how obstinate and unpractical young people were!" she thought.

Why would not Nell desire Brighton, as she herself, with her maturer judgment, did?

At length it was time for Mrs. Endsleigh and Sir Charles to go down to dinner. Rachel thought she had never known so long an afternoon. The hands of the clock seemed to crawl; the hours dragged by with leaden-footed slowness.

The afternoon dragged on. The gong sounded, and Rachel rose. It was the signal for her to go to Nell's sitting-room, and stay there while the others were at dinner. At last, her long-waited-for opportunity had arrived; she could explain to Nell her sudden change of plans, and tell her she was no longer ready to accompany them on their journey.

"I seem to have seen nothing of you to-day, nurse," said Nell, as Rachel came in. "I have been so busy. It will be better at Bedd-gelert, I shall have more time there to have you to myself. Charley will be out fishing or shooting. Here, poor boy, he has nothing to do, so comes to me to amuse him."

"Should you mind if I did not go with you to Bedd-gelert, Miss Endsleigh?" asked Rachel slowly and hesitatingly. She found it difficult to announce her change of plans to Nell, now the time had come. She realised that parting from the girl she had nursed for so many weeks would be a harder wrench than she had imagined.

"Mind!" repeated Nell in an astonished tone. "Mind! Of course I should, if such an unlikely thing were to happen. You are fishing for compliments, nurse, and ought to be ashamed of yourself! I won't satisfy your vanity, so don't ask any more questions."

"But I am quite in earnest, Miss Endsleigh. I do not wish to go to Bedd-gelert. I want you to make some arrangement for replacing me, so that I may be free."

"You are as bad as mother!" replied Nell a little pettishly. "I do believe you are afraid of the dulness. You cannot dislike the people, as you used to live in Wales."

"I do not wish to leave you," said Rachel. "I would not do so now if I did not feel it to be necessary."

"There can be no possible reason for your not going," returned Nell shortly. She was hurt, and therefore disposed to be a little unjust. That Rachel should propose leaving her—just now, too, when she so especially depended on her in case she should be ill or knocked up by the journey, seemed unreasonable.

"You were quite willing to go at first. Why should you change your mind so suddenly?"

"I cannot tell you, Miss Endsleigh," returned Rachel nervously. "Please do not ask me. But it is quite impossible I should go."

She lacked the courage to tell Nell the true reason for her refusal. She preferred seeming cold, ungrateful, and unkind, to telling the plain truth. She was so accustomed to being misunderstood and falsely judged that it seemed natural and quite in the ordinary course of events that she should be so in this case also.

Nell was surprised and disappointed; she loved Nurse Challice; and now she thought Rachel was weary of her and only eager to seize a decent excuse for leaving.

"Of course we should not think of detaining you against your will," she said coldly. "I will speak to Mrs. Endsleigh when she comes up from dinner, and she will settle what she wishes about replacing you."

She turned her head away from Rachel, and shut her eyes, as a signal that she did not wish for any further discussion on the subject.

Rachel watched her stealthily, as she lay with the soft light of the shaded lamp upon her face.

She looked very fragile, very unfit to take care of herself. Her day of unusual excitement had been too much for her slender stock of strength.

Rachel's heart smote her as she looked at the young girl, with her pale transparent features, and noted the black circles round the closed, dark eyes; saw how thin and white were the small hands clasped loosely on her white, soft silk draperies. The selfishness of her conduct rose up strongly before her. In that moment she felt the cowardice of her own behaviour; and, with a sense of shame, recognized the true reason for her dislike and dread at the thought of returning to Bedd-gelert. She knew that she shrank from seeing David, and she despised herself for her weakness.

Mrs. Endsleigh coming up after dinner saw at once that something had gone wrong.

As a rule, Nell was always particularly bright and cheerful after her quiet hour alone with Nurse Challice. This especial evening she scarcely looked up when her mother came in, and lay still without speaking.

"What is the matter?" asked Mrs. Endsleigh, turning anxiously towards Rachel. "Is not Miss Endsleigh well?"

"She seems over-tired ma'am," replied Nurse Challice. "I fancy she has done rather too much to-day, and seen too many people."

"It is not that!" exclaimed Nell, half rising from her couch. "I should have been all right but for what you said. Mother, Nurse Challice is not coming with us to Bedd-gelert."

Mrs. Endsleigh looked incredulous.

"Nonsense! The whole thing is settled. We depend on Nurse Challice; in fact, nothing would induce me to consent to going there without her. Miss Endsleigh has made a mistake, has she not, Nurse? She has misunderstood something you have said!"

She knew her daughter's quick impulsive nature, how often she said or did a thoughtless thing, repented of the next minute, and fancied that Nell must in some way have annoyed or offended the nurse.

"No, Mrs. Endsleigh," returned Rachel, in a low voice. "Miss Endsleigh is quite right. I did tell her that I should prefer your getting another nurse in my place. I would rather not go with you."

"I will have no one else," said Nell, warmly. "Unless Nurse Challice goes I will have nobody."

Mrs. Endsleigh looked infinitely distressed. Poor Rachel herself was almost crying.

"I cannot understand it," said the elder lady. "Why have you changed your mind? Has anything been done or said to make your staying with us unpleasant to you? If so, tell me at once, and I will see that everything is arranged to your satisfaction."

"No, indeed, Mrs. Endsleigh," replied Rachel quickly, "Everyone has been only too kind and considerate to me. I know I must seem ungrateful. I feel so myself, though I do not intend it. But for all that, even though I know I shall get into trouble for giving up my case, I must ask you to allow me to leave."

"But I cannot consent to your going, nurse," said Mrs. Endsleigh piteously. "You do not realize how dreadfully inconvenient, to say the least of it, such a change would be. Of course, if you have any private trouble that calls you away, we could not keep you; we should have to manage then as best we could. Are any of your relations ill? Does your family want you?"

"No," replied Rachel, "I have scarcely a relation in the world. They do not want me—nobody does."

The last bitter words slipped out unintentionally, and she was sorry the moment she had spoken them.

Nell sprang up, and took her hand.

"There you are wrong—quite wrong! Somebody does want you. I do."

Rachel looked into the beautiful pale face, and at the large dark eyes raised so pleadingly to hers.

Something seemed suddenly to remind her of Esther—of the young girl whom she had vowed to shield; to whom she had been in all things a second mother; for whom she had given up everything.

The spirit of self-sacrifice was still strong in Rachel's heart.

"Then I will go with you," she said steadily, though her grey eyes had a sorrowful, frightened look in them. "Please, Miss Endsleigh, forget what I have said. Forgive me for having been so ungrateful. I will go with you willingly to Bedd-gelert."

*(To be continued.)*

